# School climate and mental health among Swedish adolescents: a multilevel longitudinal study

**DOI:** 10.1186/s12889-019-8018-0

**Published:** 2019-12-17

**Authors:** Krisztina D. László, Filip Andersson, Maria Rosaria Galanti

**Affiliations:** 1 Department of Public Health Sciences, Karolinska Institutet, Tomtebodavägen 18A, level 3, 17177 Stockholm, Sweden; 2 Center for Epidemiology and Community Medicine, Stockholm County Council, Solnavägen 1E (Torsplan), 113 65 Stockholm, Sweden

**Keywords:** School climate, Adolescents, Internalizing problems, Externalizing problems

## Abstract

**Background:**

School is one of the most formative institutions for adolescents’ development, but whether school environment affects mental health is uncertain. We investigated the association between the school’s pedagogical and social climate and individual-level mental health in adolescence.

**Methods:**

We studied 3416 adolescents from 94 schools involved in KUPOL, a longitudinal study conducted in eight regions in Sweden. School climate was reported by the school’s teaching personnel and by the final year students using the teacher and the student versions of the Pedagogical and Social Climate Questionnaire, respectively. Index persons’ mental health was assessed with the Center for Epidemiological Studies Depression Scale for Children and the Strengths and Difficulties Questionnaire. We performed multilevel logistic regression models adjusted for individual, familial and school-level confounders measured in grade 7 and exposure and outcome measured in grades 8 and 9.

**Results:**

The adjusted odds ratios and 95% confidence intervals comparing the middle and the high to the lowest tertile of the total teacher school climate score were 1.47 (1.10–1.97) and 1.52 (1.11–2.09) for depressive symptoms and 1.50 (1.08–2.08) and 1.64 (1.16–2.33) for the total strengths and difficulties score. In contrast, there was no association between total student school climate score and mental health.

**Conclusions:**

We found that teacher-, but not student-rated school climate was associated with an increased risk of poor mental health at the student level; the association was most pronounced for internalizing problems. Given schools’ importance for adolescents’ development, further studies are needed to clarify the mechanisms underlying the observed association.

## Background

The increase in rates of several mental health problems in adolescents and young adults in Western countries represents an important public health concern. Mental health problems were the main cause of disability-adjusted life years in the age group 10–24 years in middle- and high-income countries in 2017, increasing their rank by three positions compared to 1990 [1]. Following this evidence, the World Health Organisation has endorsed this problem as a priority agenda for the EU region [2, 3]. In Sweden, the increase in poor mental health in youths has been particularly steep in an international perspective [4], has concerned primarily mood and psychosomatic disorders and suicide attempts [5], and has affected girls to a larger extent than boys [5].

The Swedish government [5] and the Public Health Agency of Sweden [6] commissioned extensive analyses to elucidate the mechanisms behind the rising trends of poor mental health among Swedish youths on several occasions. Factors related to school and to perceived requirements to enter the labour market were the strongest potential explanations for the increase in rates of poor mental health among Swedish youths, prompting initiatives to improve the school and the learning environments [5, 6]. Interestingly, a similar British investigation also concluded that changes related to school were among the most important contributors to the rising trends of poor mental health among youths [7]. The Swedish education system underwent substantial changes after the severe economic crisis that hit the country in the early 1990s. The most influential reforms concerned (1) the shift of the responsibility for education from the national to the municipal level; (2) the establishment, in parallel to the public school system, of privately run, but publicly funded schools; (3) free school choice; (4) autonomy for schools and teachers in shaping the content and the methods of teaching to attain the centrally set educational goals and (5) a shift towards a decrease in the teachers’ and an increase in the student’s responsibility for learning [8]. The organisation of education is divided between (1) the central government, which is responsible for setting the national educational goals, developing the curriculum and monitoring the performance of the educational system, and (2) the municipalities, which together with the individual schools (public or private) are responsible and accountable for organising education in a manner that ensures meeting the national educational goals [8]. Schools have autonomy in interpreting the national curriculum and in the choice of educational methods. Private schools tend to be more specialised with regard to pedagogical orientation and subjects offered (e.g., religion, arts, foreign languages etc.) than municipal schools [8]. Students with special educational needs (including those with mental illness) generally attend usual classes, but they may attend special schools if necessary [9].

The potential consequences of the mentioned major reforms – when during a few years Sweden’s education system turned from being one of the most centralized in the OECD to be one of the most decentralized – have fuelled extensive discussions [8], some of which may also be relevant to other Western countries given increasing considerations about more inclusive education and about enacting shifts towards its decentralisation [9]. On the one hand, the reforms stimulated diversity and competition among schools and have increased local autonomy [8]. On the other hand, the shift of the responsibility for education from the central to the municipal and subsequently to the school level resulted in a defragmentation of the educational responsibility [8, 9]. School results declined for Swedish students at all levels of academic performance; the low-performing students experienced the most pronounced decline [8, 10]. Social segregation and inequalities in academic results among schools have increased [9]. The culture, norms, pedagogical climate, the emphasis the individual schools put on academic achievement and the support the schools provide to reach the educational goals have thus become increasingly important for Swedish students’ academic results; to what extent such aspects of the school are related to their students’ well-being is not clear. Though there is no consensus with respect to the definition of school climate, most reviews in this area include the domains (1) academic climate, (2) community, (3) safety and discipline and (4) physical environment [11]. Increasing evidence suggests that self-perceived positive characteristics of the climate of a school are associated with mental health [12]. However, whether these associations are causal is not clear as most of the studies in this field were cross-sectional [13–20], did not use multilevel design to separate the effect of the school climate from that of their student composition and relied on aggregation of self-reported exposure from the persons whose mental health was assessed [13–21]. The three longitudinal multilevel studies in this area yielded mixed results. A large Canadian study, assessing exposure and outcome with different student informants, reported an inverse association between the quality of the school’s socioeducational environment and the risk of depressive symptoms at follow-up [22]. In the other two longitudinal multilevel studies in this area the same group of students were informants on contextual school climate and individual-level mental health; Joyce & Early found an inverse association between school connectedness and teacher support at baseline and depressive symptoms at follow-up [21], whereas Winfree and Jiang found no association between school support and later suicide ideation or attempt [21]. Most of the previous studies in this area did not assess school climate systematically, but focused on a limited number of school domains [13, 15–18, 20, 21, 23]; few studies included measures of externalizing problems [19]. None of the previous investigations analysed school climate as perceived by teachers.

An important underlying mechanism for the association between a school climate and mental health is that a good school climate may foster academic achievement, which in turn may predict mental health [24]. The link between school failure and the risk of internalizing (e.g. depression, anxiety or self-harm) and externalizing mental health problems (e.g. hyperactive, attention or conduct disorders) is well-established [24]. On the other hand, the pressure related to academic achievement is regularly named by Swedish adolescents as one of their most important sources of stress [5, 24]; performance pressure is in turn associated with an increased risk of internalizing problems [24]. Similarly, other aspects of the school climate, e.g. evaluation, feedback, discipline, responsibility and free choice, may contribute differently to specific mental health problems depending on how they are implemented by the school and how they are perceived by the students.

We aimed to investigate the association between school climate using reports from multiple informants and individual-level mental health in a large longitudinal cohort of Swedish adolescents.

## Methods

### Study population and design

**Fig. 1:**
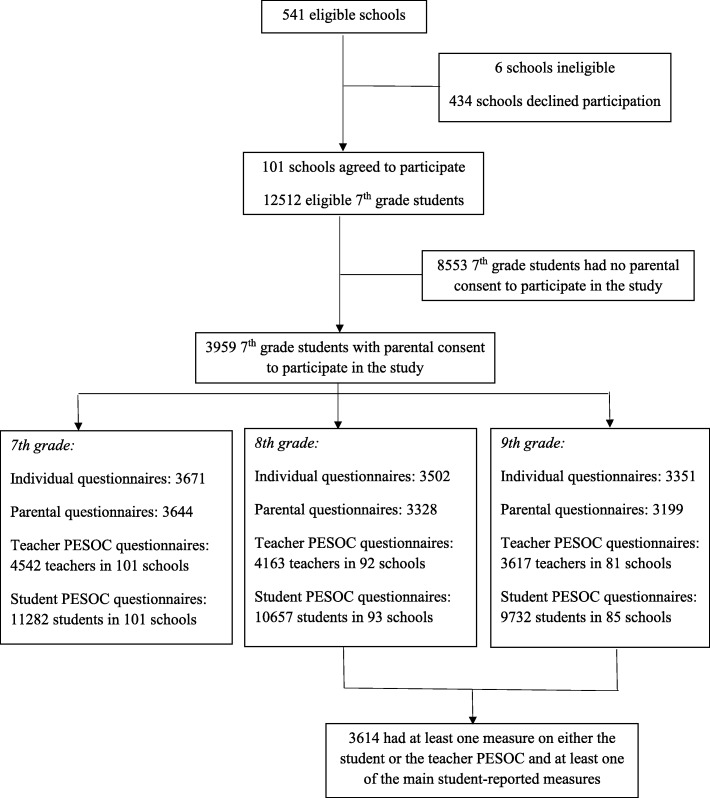
Flow chart for participation in the study

We studied adolescents involved in the longitudinal KUPOL (Swedish acronym for “Knowledge about Adolescents Mental Health and Learning”) study [25]. Shortly, 541 secondary schools located in eight Swedish regions, with at least 20 students per year in grades 7–9, were invited to participate in the study in 2013. The 101 consenting schools forwarded written information about the study to their 7th grade students and to their guardians in the 2013/2014 and in the 2014/2015 academic years. Of the 12,512 eligible adolescents, the guardians of 3959 pupils provided written informed consent for the adolescent’s participation in the study. Data collection involved multiple informants during grades 7, 8 and 9. Information on adolescents was obtained from self-reported questionnaires and questionnaire filled in by parents. Information on school-level factors was obtained from (1) questionnaires completed by the schools’ teaching personnel, (2) questionnaires completed by the schools’ 9th grade students and (3) the statistical databases of the Swedish National Agency for Education. The flow chart of participation in the study is shown in Fig. 1. Analyses for the present study were restricted to students who had information on at least one main exposure measure and on at least one of the main student-reported mental health scales (see next section) in either grade 8 or 9 (*n* = 3416).

### Measures

The *schools’ pedagogic and social climate* was assessed using the teacher and the student version of the Pedagogical and Social Climate (PESOC) Questionnaire [26–28]. *Mental health* was ascertained using the Center for Epidemiological Studies Depression Scale for Children (CES-DC) and the self-reported and parent versions of the Strengths and Difficulties Questionnaire (SDQ). More information about these four questionnaires is provided in Additional file 1.

Several *potential confounders* were chosen based on (1) their potential association with school climate / the students’ choice of school and with mental health and (2) not being on the causal pathway between school climate and mental health. Information on parents’ country of origin and education and on the adolescent’s gender were obtained from the baseline questionnaires and were categorized as shown in Table 1. In case information on these variables were missing at the first assessment we used information from the second or third assessment. Parental cognitive school engagement was assessed with the ‘Future Aspirations and Goals’ subscale of the Student Engagement Instrument [29], described in Additional file 1. Information on school-level factors, i.e. school ownership (municipal or private school), percentage of parents with post-high school education, parents born abroad and teachers with a pedagogical university degree at the school level – was retrieved from the SIRIS database of the National Agency for Education. We also recorded whether the school was in a rural or an urban area.

**Table 1:** Characteristics of the sample according to student and teacher PESOC tertiles

Variable	N	Total teacher PESOC tertile in wave 2				Total student PESOC tertile in wave 2			
		Low (*n* = 1093)	Middle (*n* = 1084)	High (*n* = 1168)	*p*-value^a^	Low (*n* = 1029)	Middle (*n* = 1118)	High (*n* = 1212)	*p*-value^a^
Categorical variables		%	%	%		%	%	%	
School ownership	3416				< 0.001				< 0.001
Municipal school		92.31	69.28	47.09		87.46	78.80	43.73	
Private school		7.69	30.72	52.91		12.54	21.20	56.27	
Gender	3416				0.23				0.22
Boy		49.04	46.31	49.74		50.34	48.84	46.70	
Girl		50.96	53.69	50.26		49.66	51.16	53.30	
Parental education	3360				< 0.001				< 0.001
No parent with post-high school education		36.85	26.34	25.88		30.16	35.10	24.12	
At least one parent with post-high school education		63.15	73.66	74.13		69.84	64.90	75.88	
Parental country of origin	3186				0.32				0.048
No parent born abroad		80.57	78.97	81.57		80.41	78.25	82.46	
At least one parent born abroad		19.43	21.03	18.43		19.59	21.75	17.54	
School’s geographical location	3416				< 0.001				< 0.001
Rural		67.6	44.37	42.89		55.69	64.58	35.15	
Urban		32.4	55.63	57.11		44.31	35.42	64.85	
Continuous variables		Median	Median	Median		Median	Median	Median	
% of parents with post-high school education at the school level	3231	51.00	63.00	66.00	< 0.001	51.00	53.00	67.00	< 0.001
% of parents born abroad at the school level	3104	17.00	14.00	11.00	< 0.001	14.00	15.00	14.00	< 0.001
% of teachers with pedagogical university degree at the school level	3237	85.20	83.70	80.90	< 0.001	82.90	83.80	83.50	< 0.001
Parental school engagement	3122	23.00	24.00	24.00	0.013	23.00	23.00	24.00	0.0015
CES-DC in grade 7	3212	12.00	12.00	12.00	0.58	12.00	12.00	12.00	0.86
Total self-reported SDQ in grade 7	3225	9.00	9.00	9.00	0.21	9.00	9.00	9.00	0.43
Total parent-reported SDQ in grade 7	3135	5.00	4.00	5.00	0.14	4.00	5.00	4.00	0.0019

PESOC, Pedagogical and Social Climate Questionnaire; CES-DC, Center for Epidemiological Studies Depression Scale for Children; SDQ, Strengths and Difficulties Questionnaire

^a^The *p*-value corresponds to chi-square tests in case of categorical data and to Kruskal-Wallis tests in case of continuous variables

### Statistical analyses

#### Descriptive analyses

The tertiles constituted based on the PESOC total scores were compared on covariates using chi-square tests in case of categorical variables and Kruskal-Wallis tests in case of continuous variables. The relation between the continuous teacher and the student PESOC scale was investigated using Pearson correlation. The association between baseline covariates and the risk of poor mental health was analysed by means of the SAS GLIMMIX procedure.

#### Main analyses

Similarly, we analysed the association between school climate and mental health using the GLIMMIX procedure; we considered the clustering of the data in schools and in individuals. We used information from grade 7 on potential confounders and from grade 8 and 9 on exposures and outcomes. We first ran empty models with each of the four main outcomes and calculated the intra-class correlations using the formula intra-class correlation coefficient = covariance parameter estimate/(covariance parameter estimate + 3.29), as suggested by Ene et al. [30]. Next, we ran several models relating tertiles of the total teacher and student PESOC scores to each of the four main outcome measures. Model 1 was unadjusted. Model 2 was adjusted for baseline mental health and demographic factors, i.e. adolescent’s gender, parental education, parental country of origin, school ownership and geographical location. To address concerns related to the short time interval between measurements, which may make disentangling bidirectional associations between some confounders and school climate difficult, we added parental cognitive school engagement and school-level demographics (percentage of parents with post-high school education, percentage of parents born abroad and percentage of teachers with pedagogical university degree at the school level) in a separate model (Model 3). Subsequently, we conducted multivariate analyses with the subscales of the teacher and the student PESOC questionnaires.

#### Sensitivity analyses

Given the higher rate of adolescents with Swedish born and highly educated parents in our sample compared to the target population [25], we performed stratified analyses by parental education and country of origin to investigate whether these factors may modify the association between total PESOC and the primary outcomes. In further sensitivity analyses we re-ran the models concerning the association between total PESOC and mental health after excluding schools that had a response rate for PESOC below 30%. We also repeated the analyses concerning the association of student total PESOC with the three parent-reported mental health outcomes, i.e. SDQ total score, SDQ internalizing problems and SDQ externalizing problems.

Analyses were performed using SAS 9.4.

## Results

### Descriptive analyses

The proportion of private schools, urban schools, parents with post-high school education and of the teachers without a university degree at the school level increased with increasing student and/or teacher PESOC tertiles; parental school engagement was higher in the two upper than in the lowest teacher PESOC tertile (Table 1). The associations between baseline covariates and poor mental health at follow-up are presented in Additional file 1: Table S2. The correlation coefficient between the continuous teacher and student total PESOC score was 0.48 (*p* < 0.001) in both wave 2 and 3.

### Main analyses

The school level intra-class correlation was 2.12% for depressive symptoms, 4.38% for SDQ total problems, 2.46% for internalizing problems and 2.11% for externalizing problems. After adjustment for CES-DC/total SDQ score in grade 7, the adolescent’s gender, parental education, parental country of origin, school ownership and geographical location, the total teacher PESOC score was associated with an increased risk of high SDQ total problems and internalizing problems score (Table 2). In contrast, there was no association between total student PESOC score and the four indicators of mental health. These associations did not change substantially after further adjustment for parental cognitive school engagement, percentage of parents with post-high school education, percentage of parents born abroad and percentage of teachers with pedagogical university degree at the school level (Table 2). Most subscales of the teacher PESOC were or tended to be associated with an increased risk of high CES-DC and high total SDQ score (Additional file 1: Figure S1a, b and Table S3); similar trends were observed also for SDQ internalizing and externalizing problems, though with less power (Additional file 1: Figure S1c, d and Table S3). In analyses with the subscales of the student PESOC, we observed an association only between (1) school environment and (2) student participation and an increased SDQ total problems score (Additional file 1: Figure S2 and Table S3).

**Table 2:** Odds ratios for poor self-reported mental health according to school pedagogical and social climate

Measure of mental health	Poor mental health (%)^a^		Model 1 OR (95% CI)	Model 2^b^ OR (95% CI)	Model 3^c^ OR (95% CI)
	grade 8	grade 9			
Teacher PESOC score tertile					
CES-DC					
Lowest	10.46	13.77	1.00	1.00	1.00
Middle	13.11	15.19	1.24 (0.98–1.57)	1.47 (1.10–1.97)	1.47 (1.06–2.02)
High	13.31	16.83	1.31 (1.03–1.67)	1.52 (1.11–2.09)	1.41 (1.01–1.98)
SDQ total problems					
Low	10.07	11.84	1.00	1.00	1.00
Middle	10.63	12.93	1.20 (0.91–1.58)	1.50 (1.08–2.08)	1.42 (0.99–2.05)
High	12.16	14.00	1.34 (1.01–1.77)	1.64 (1.16–2.33)	1.64 (1.12–2.40)
SDQ internalizing problems					
Low	13.33	15.88	1.00	1.00	1.00
Middle	14.44	16.89	1.14 (0.90–1.44)	1.28 (0.96–1.70)	1.40 (1.02–1.91)
High	16.31	18.24	1.32 (1.04–1.68)	1.48 (1.09–2.00)	1.45 (1.05–2.01)
SDQ externalizing problems					
Lowest	7.86	8.90	1.00	1.00	1.00
Middle	8.14	8.59	1.10 (0.83–1.47)	1.34 (0.97–1.85)	1.23 (0.86–1.76)
High	9.31	8.37	1.15 (0.86–1.54)	1.27 (0.90–1.79)	1.23 (0.84–1.78)
Student PESOC score tertile					
CES-DC					
Low	12.30	13.77	1.00	1.00	1.00
Middle	11.90	16.94	1.14 (0.92–1.41)	1.21 (0.93–1.56)	1.23 (0.93–1.63)
High	12.57	14.07	0.98 (0.77–1.25)	0.95 (0.70–1.28)	0.90 (0.65–1.25)
SDQ total problems					
Low	10.65	12.94	1.00	1.00	1.00
Middle	11.47	13.58	1.11 (0.88–1.40)	1.10 (0.83–1.45)	1.22 (0.89–1.66)
High	10.52	11.54	1.02 (0.78–1.34)	1.04 (0.75–1.45)	1.12 (0.78–1.61)
SDQ internalizing problems					
Low	13.77	16.74	1.00	1.00	1.00
Middle	15.13	17.77	1.11 (0.90–1.37)	1.11 (0.87–1.42)	1.16 (0.89–1.52)
High	15.00	15.89	1.03 (0.82–1.30)	1.00 (0.75–1.33)	0.98 (0.72–1.33)
SDQ externalizing problems					
Low	9.13	10.18	1.00	1.00	1.00
Middle	7.89	8.45	0.88 (0.69–1.14)	0.86 (0.64–1.16)	0.84 (0.60–1.16)
High	8.23	7.20	0.88 (0.66–1.16)	0.96 (0.69–1.34)	0.96 (0.67–1.38)

*OR* odds ratio, *CI* confidence intervals, *CES-DC* Centre for Epidemiological Studies Depression Scale for Children, *SDQ* Strengths and Difficulties Questionnaire

^a^Subjects with missing data are excluded

^b^Adjusted for school ownership, the school’s geographical location, adolescent’s gender, CES-DC/SDQ score in grade 7, parental education and parental country of origin

^c^Includes besides the variables in model 2 parental cognitive school engagement, percentage of parents with post-high school education, percentage of parents born abroad and percentage of teachers with pedagogical university degree at the school level

### Sensitivity analyses

The associations between teacher and student total PESOC score and poor mental health did not differ substantially according to parental education and parental country of origin. Repeating the main analyses after excluding schools where the response rate on the teacher and student PESOC was below 30% did not change the results considerably. The association between the teacher and the student total PESOC scores and the parent-reported total SDQ score and internalizing and externalizing problems were substantially more modest than those observed using self-reported mental health (Additional file 1: Table S4).

## Discussion

A positive school climate as perceived by its teachers was associated with an increased risk of poor mental health, primarily of internalizing problems; there was a similar or a trend toward a similar association for most of the dimensions of the teacher-perceived school climate. The associations between student-reported overall school climate and its specific dimensions and the risk of poor mental health were substantially more modest, if at all present.

### Comparison with previous studies and potential explanations for our findings

Emerging, though not consistent, evidence suggests that self-perceived characteristics of the climate of a school – e.g. school connectedness, teacher and peer relationships, safety, fairness etc. – are positively associated with mental health [12]. However, it has been argued that school climate is a contextual construct and, as such, it should be assessed at the school level [11, 28] and that individual-level studies in this field are prone to confounding by cognitive biases specific to depression, (e.g., a tendency to assess negatively both school climate and mental health) [22]. With a few exceptions [21–23], studies in this field had a cross-sectional design [13–20], thus reverse causation could further contribute to the observed associations. Few studies separated the effect of school climate from that of its student composition by multilevel design, most had the same group of students as informants on exposure and outcome [13–20] and very few assessed school climate systematically [13–18, 20]. Two longitudinal multilevel studies, one assessing exposure and outcome with different informants [22] (an approach that may further limit unmeasured confounding [11, 12]) and one using the same group of students as informants on contextual school climate and individual-level mental health [23], found inverse associations between the school’s socioeducational environment [22] and school connectedness and teacher support [23] at baseline and depressive symptoms at follow-up. In contrast, Winfree and Jiang found no association between school support and later suicide ideation or attempt [21]. Our study extends the evidence concerning the relation between school climate and mental health by using a multilevel longitudinal design, assessing both exposure and outcome with independent and multiple informants with validated questionnaires, focusing on both internalizing and externalizing problems, and considering a wide range of individual- and school-level factors assessed prior to exposure. The differences in study design are likely to be important explanations for the discrepancy between the results observed in our study and those of several earlier studies reporting an association between positive dimensions of school climate and good mental health. Though the use of independent student informants may avoid spurious associations due to confounding by negative affectivity or due to reverse causation, it has the potential disadvantage of poor relevance of the exposure for the index cohort, i.e. 9th grade students who rated the climate of the school may ignore problems that are relevant to the actual cohort, thus attenuating a potential association. Furthermore, in contrast to most previous studies in this area, which had an important focus on school connectedness and school-based social relationships [17, 21, 23], a strong emphasis in the PESOC instruments was on the pedagogical climate, a discrepancy which may have further contributed to differences in findings between our study and previous studies in this field.

To our knowledge this is the first study to investigate the association between teacher-reported school climate and students’ mental health. Our findings that the teacher-reported positive school climate was associated with an increased risk of poor mental health among students is somewhat intriguing. The fact that a similar association or a trend toward a similar association was observed between most of the scales of the teacher PESOC and mental health (primarily internalizing problems) suggests that a set of common factors may underlie these relationships. Several studies among Swedish adolescents highlight that one of their most prominent sources of stress is school-related demands and pressure [5, 24] and that this may increase the risk of internalizing problems [24]. Though several of the school characteristics assessed by the teacher PESOC questionnaire may be regarded as positive in themselves, we speculate that schools with higher teacher-reported PESOC scores may put more pressure on their students to achieve good results than schools with lower PESOC scores. Although a certain level of demands and pressure is beneficial, an imbalance between external demands and the students’ abilities to handle them induces strain and may increase the risk of poor mental health. While for a substantial proportion of pupils, there is a decrease in school-related motivation in adolescence [31, 32], school demands and perceived school-related pressure increase during this life period. The steep increase in perceived school pressure between the ages 11 and 15 among Swedish adolescents and their higher school pressure at the age of 15 compared to the corresponding European mean [32] may be related to the late introduction of academic grades in Sweden [15] and the more pronounced negative time trends concerning young adults’ possibilities to enter the labour market in Sweden than in other European countries [4]. Swedish primary schools emphasize self-improvement, i.e. “mastery goal orientation”, whereas competition, i.e. “relative ability orientation” [31] is discouraged. However, the introduction of the academic grades in grade 6 and their importance for subsequent education inevitably stimulates comparisons among peers. Although generally there is no academic tracking in Swedish primary schools, the free school choice, the importance of parental education and time since immigration for informed school choice and the residential segregation favour some grouping of pupils according to their parents’ academic background. According to the “big-fish-little-pond effect” from social comparison and self-concept theories, same ability students tend to lower their academic self-concept in higher average ability schools, and tend to increase it in lower average ability schools [33]. Though attending schools with high PESOC score – having on average better academic results [27] – may enhance health on the long-term through higher educational achievements, it is possible that the propensity toward upward comparison and the decreased academic self-concept in schools with high PESOC scores may increase the risk of internalizing problems on the short term. This effect could be further enhanced by a school context in which pupils in early adolescence are not used to peer comparisons, and by an age when an increasing number of pupils realize that entering higher education and the labour market involves competition, and when their knowledge about reference frames larger than that of their schools is limited [33]. The differences in knowledge about national reference frames between the adolescents and their parents might partly explain differences in the association of teacher PESOC with self- and parent-reported pupil mental health.

We observed only a moderate correlation between the teacher and the student versions of the PESOC questionnaire, suggesting that there are differences in the aspects they capture. In addition to teaching activities and social relationships included in both questionnaires, the teacher version also assesses school management and pedagogical leadership. A further, rather subtle, difference between the two questionnaires is that the items of the student PESOC are often formulated in terms of the support and resources that students receive at school, whereas the formulation of the teacher PESOC tends to put more emphasis on expectations, school rules and norms, further supporting the school-related demands as a potential explanation for the association between teacher, but not student PESOC. Education is an important cue for cognitive and socioemotional development and achieved education is one of the most important predictors of health over the lifecourse [34]. Correspondently, several public health agencies have started to recognize the importance of school not only as a learning environment, but also as an environment for prevention, early detection and management of psychological distress among children and adolescents [3]. Nevertheless, a report recently published by the World Health Organisation recognizes that although “*health and academic attainment need to go hand in hand”* they *“are often addressed separately”* [3] (p. 94) and that this separation may represent a challenge for effectively implementing public health interventions. Our finding of an increased risk of poor mental health among schools with high teacher-reported PESOC scores – which on average have better academic results [27] – may be reflective of such difficulties in inter-sectorial cooperation and calls for better coordination between the education and the health sectors to promote high educational achievement, while maintaining good mental health [3].

### Limitations

A limitation of our study is that despite intensive efforts to increase participation in the study, enrolment rate at the school and at the individual level was modest and adolescents of Swedish-born and highly educated parents are overrepresented in our sample compared to the target population [25]. Whether this selection affected our results is unclear; results from our stratified analyses suggest no differences in the investigated associations according to parental education and country of origin. Second, though we adjusted for a large number of potential confounders we cannot exclude the possibility of residual confounding, e.g. from parental mental health and socioeconomic factors at the municipality level.

## Conclusions

We found that teacher-, but not student-rated school climate was associated with an increased risk of poor mental health, primarily internalizing problems, among the students. Given the important formative role schools have on adolescents’ development and the potential public health implications of our findings, studies that would contribute to a better understanding of the mechanisms underlying the observed associations are needed. In particular, studies that would contribute to a better understanding of the relationship between school emphasis on academic achievements and adolescents’ emotional development are warranted. Our findings might be suggestive of a need of increased collaboration between the education and the health sectors to promote high educational achievement, while maintaining good mental health [3].

## Supplementary information

**Additional file 1.** Supplementary materials.

## Data Availability

The datasets generated and/or analysed during the current study are not publicly available in order to protect the privacy of study participants and to comply with the content of the ethical application, but are available from the corresponding author on reasonable request.

## Abbreviations

CES-DC: Center for Epidemiological Studies Depression Scale for Children
KUPOL: Swedish acronym for “Knowledge about Adolescents Mental Health and Learning”
PESOC: Pedagogical and Social Climate
SDQ: Strengths and Difficulties Questionnaire
