# Supplementary material for: School climate and mental health among Swedish adolescents: a multilevel longitudinal study
Source: BMC Public Health. 2019 Dec 17;19:1695. doi: 10.1186/s12889-019-8018-0 (PMC6918582; doi:10.1186/s12889-019-8018-0)

# Supplementary material

**Detailed description of the scales used in the study**

*The teacher version of the Pedagogical and Social Climate (PESOC) Questionnaire* was given for completion to all teaching personnel in the participating schools [1]. The questionnaire consists of 59 items that assess teachers’ perceptions of the following dimensions of their school: (1) Teachers expectations for students’ behavior and academic performance (4 items), (2) Perceived teacher agreement about school goals, norms and rules (9 items), (3) Student focus (6 items), (4) Basic assumptions about students’ ability to learn (3 items), (6) Communication between school and home (5 items), (5) Teacher interaction and cooperation (4 items), (6) Teachers’ confidence and professional development (5 items), (7) Teaching activities (6 items), (8) Evaluation of students’ academic progress (2 items), (9) Principal’s pedagogical leadership (12 items) and (10) Teacher’s perceptions of school management’s involvement and support (3 items) [1].

*The student version of the PESOC* [2] was given to all 9^th^ grade students in the school since they spent the longest time in school and were considered to be the most familiar with the ethos of the school. The questionnaire consists of 53 items and assesses the students’ perceptions of the following eight dimensions of their school: (1) Expectations (4 items), (2) Perception of teacher norms (12 items), (3) Teachers’ support (5 items), (4) Teaching activities (15 items), (5) Student participation (3 items), (6) School environment (3 items), (7) School and home (5 items), and (8) School management (6 items) [2].

In both questionnaires, response alternatives were given on a four-point Likert scale; higher scores reflect more favorable school characteristics. The total and subscale-specific scores were calculated as the average of the responses by teachers in the teacher version and 9^th^ grade students in the student version of the PESOC Questionnaire. A “do not know” response alternative was also included, which was used only for statistical purposes [1, 2]. Since in case of several subscales the range was smaller than 1 (Table S1), we categorized the total scores and the scores on the specific subscales based on their tertile distribution. Both questionnaires were found to have good psychometric properties in the validation studies based on KUPOL or its pilot study [1, 2]. Reliability coefficients for each subscale based on answers from wave 2 are presented in Table S1. The items of the two version of the questionnaire have been presented by Hultin et al. [1, 2].

*The Center for Epidemiological Studies Depression Scale for Children* is a 20-item questionnaire that inquired adolescents about the frequency of depressive symptoms in the past week [3]. Responses are given on a four-step Likert scale; the total score is obtained by summing the responses on the individual items. We dichotomized the variable as <30 versus ≥30, as previously suggested [3]. The scale’s Cronbach alpha in wave 2 was 0.90.

*The Strengths and Difficulties Questionnaire* includes 25 items that inquire about emotional symptoms, hyperactivity/inattention symptoms, conduct problems, peer problems and prosocial behavior [4]. We generated (1) a total problem score by summing the responses on the 25 items, (2) the internalizing problem score by summing scores on the emotional symptoms and the peer problems scales and (3) the externalizing problem score by summing answers on the conduct problems and the hyperactivity/inattention scales [4].We dichotomized the scales based on the following suggested cutoffs: (1) self-reported total difficulties score <18 versus ≥18; (2) self-reported internalizing problems score <9 versus ≥9; (3) self-reported externalizing problems score <11 versus ≥11; (4) parent-reported total difficulties scores <17 versus ≥17; (5) parent-reported internalizing problems score <8 versus ≥8; (6) parent-reported externalizing problems score <11 versus ≥11 [4, 5]. Cronbach alphas for the six SDQ scales (self- and parent-reported total SDQ, internalizing and externalizing problems scores), in wave 2 ranged between 0.72 and 0.82.

*The ‘Future Aspirations and Goals’ subscale of the Student Engagement Instrument* [6]*,* contains five items that ask parents about the value they put on their adolescent’s education, the importance of continuing education and their hopefulness in this respect. The total score is calculated as the mean of the responses on the five items. The Cronbach alpha at wave 2 was 0.74.

**References**

1. Hultin H, Ferrer-Wreder L, Eichas K, Karlberg M, Grosin L, Galanti MR: **Psychometric Properties of an Instrument to Measure Social and Pedagogical School Climate among Teachers (PESOC).** *Scandinavian Journal of Educational Research* 2018; **62**:287-306.
2. Hultin H, Ferrer-Wreder L, Eichas K, Karlberg M, Grosin L, Galanti MR: **Pedagogical and Social School Climate: Psychometric Evaluation and Validation of the Student Edition of PESOC.** *Scandinavian Journal of Educational Research* 2019; **63**:534-550.
3. Olsson G, von Knotting AL. **Depression among Swedish adolescents measured by the self rating scale Center for Epidemiology Studies - Depression Child (CES-DC).** *European Child and Adolesctent Psychiatry* 1997; **6**:81-87.
4. Goodman A, Lamping DL, Ploubidis GB. **When to Use Broader Internalising and Externalising Subscales Instead of the Hypothesised Five Subscales on the Strengths and Difficulties Questionnaire (SDQ): Data from British Parents, Teachers and Children.** *Journal of Abnormal Child Psychology* 2010; **38**:1179-91.
5. Strengths and Difficulties Questionnaire, UK: http://www.sdqinfo.org/py/sdqinfo/c0.py. Accessed Jul 16, 2018.
6. Betts J, Reschly, AL, Appleton, JJ, Christenson, SL, Hueber, ES. **A study on the factorial invariance of the Student Engagement Instrument (SEI): Results from middle and high school students.** *School Psychology Quarterly* 2002; **25**:84-93.

**Figure legends**

**Figure S1** Odds ratios and 95% confidence intervals for (1) depressive symptoms, (2) total strengths and difficulties (SDQ) score, (3) SDQ internalizing problems and (4) SDQ externalizing problems comparing the middle (T2) and the higher tertiles (T3) of the teacher-reported Pedagogical and Social Climate Questionnaire scales to the lowest tertile. Adjustment was made for school ownership, the school’s geographical location, adolescent’s gender, mental health in grade 7, parental education and parental country of origin.

**Figure S2** Odds ratios and 95% confidence intervals for (1) depressive symptoms, (2) total strengths and difficulties (SDQ) score, (3) SDQ internalizing problems and (4) SDQ externalizing problems comparing the middle (T2) and the higher tertiles (T3) of the student-reported Pedagogical and Social Climate Questionnaire scales to the lowest tertile. Adjustment was made for school ownership, the school’s geographical location, adolescent’s gender, mental health in grade 7, parental education and parental country of origin.

**Supplementary tables**

**Table S1** Descriptive statistics and reliability coefficients for the subscales of the Pedagogical and Social Climate Questionnaires

| PESOC subscale | Range | Mean (standard deviation) | Cronbach alpha coefficient |
| --- | --- | --- | --- |
| Teacher PESOC |  |  |  |
| Total teacher PESOC | 2.49-3.73 | 3.24 (0.19) |  |
| Basic assumptions about students’ ability to learn | 2.90-3.85 | 3.47 (0.15) | 0.61 |
| Evaluation of students’ academic progress | 2.39-3.81 | 3.14 (0.24) | 0.78 |
| Teachers’ expectations for students’ behavior and academic performance | 2.90-3.88 | 3.51 (0.19) | 0.62 |
| Communication between school and home | 2.57-3.80 | 3.40 (0.17) | 0.72 |
| Perceived teacher agreement about school goals, norms and rules | 2.72-3.78 | 3.30 (0.19) | 0.82 |
| Principal’s pedagogic leadership | 1.85-3.86 | 2.95 (0.38) | 0.90 |
| Student focus | 2.99-3.79 | 3.47 (0.14) | 0.67 |
| Teachers’ perception of school managements’ involvement and support | 1.81-4.00 | 3.07 (0.43) | 0.91 |
| Teaching activities | 2.71-3.74 | 3.26 (0.20) | 0.67 |
| Teacher interaction and cooperation | 2.88-3.86 | 3.46 (0.19) | 0.79 |
| Teachers’ confidence and professional development | 2.69-3.84 | 3.37 (0.20) | 0.77 |
| Student PESOC |  |  |  |
| Total student PESOC | 2.48-3.48 | 2.97 (0.15) |  |
| Expectations | 3.16-3.81 | 3.53 (0.10) | 0.42 |
| Perception of teacher norms | 2.47-3.61 | 3.10 (0.17) | 0.80 |
| School environment | 1.87-3.65 | 2.70 (0.31) | 0.51 |
| School and home | 2.74-3.67 | 3.20 (0.17) | 0.67 |
| School management | 1.58-3.61 | 2.51 (0.38) | 0.77 |
| Student participation | 1.78-3.32 | 2.47 (0.24) | 0.53 |
| Teaching activities | 2.55-3.41 | 2.95 (0.14) | 0.82 |
| Teachers’ support | 2.52-3.51 | 3.06 (0.16) | 0.60 |

PESOC, Pedagogical and Social Climate Questionnaire.

**Table S2** Univariate odds ratios for poor mental health at follow-up according to the study covariates in grade 7

| Variable | OR (95% CI)^a^ | |
| --- | --- | --- |
|  | CES-DC | Total SDQ |
| School ownership  Municipal school  Private school | 1  1.13 (0.91-1.41) | 1  1.04 (0.80-1.36) |
| Gender  Boy  Girl | 1  6.48 (5.24-8.01) | 1  2.09 (1.74-2.52) |
| Parental education  No parent with post-high school education  At least one parent with post-high school education | 1  0.80 (0.67-0.97) | 1  0.54 (0.45-0.65) |
| Parental country of origin  No parent born abroad  At least one parent born abroad | 1  1.01 (0.81-1.27) | 1  1.09 (0.84-1.38) |
| School’s geographical location  Rural  Urban | 1  1.18 (0.96-1.45) | 1  1.05 (0.81-1.35) |
| % of parents with post-high school education at the school level | 1.01 (1.00-1.01) | 1.00 (0.99-1.01) |
| % of parents born abroad at the school level | 1.00 (0.99-1.01) | 1.00 (0.99-1.02) |
| % of teachers with pedagogical university degree at the school level | 1.00 (0.99-1.01) | 1.00 (0.99-1.01) |
| Parental school engagement | 0.96 (0.92-1.00) | 0.90 (0.87-0.94) |
| CES-DC in grade 7 | 1.12 (1.11-1.13) | 1.10 (1.09-1.11) |
| Total self-reported SDQ in grade 7 | 1.19 (1.17-1.21) | 1.27 (1.24-1.29) |
| Total parent-reported SDQ in grade 7 | 1.07 (1.05-1.09) | 1.13 (1.11-1.15) |

OR, odds ratio; CI, confidence intervals; CES-DC, Center for Epidemiological Studies Depression Scale for Children; SDQ, Strengths and Difficulties Questionnaire.

a: 1 indicates the reference category.

**Table S3** Odds ratios and for self-reported mental ill-health according to dimensions of school climate in fully adjusted models

| PESOC subscale | OR (95% CI)^a^ | | | |
| --- | --- | --- | --- | --- |
|  | CES-DC | SDQ total problems | SDQ internalizing problems | SDQ externalizing problems |
| Teacher PESOC |  |  |  |  |
| Basic assumptions about students’ ability to learn | | | | |
| Medium  High | 1.12 (0.83-1.50)  1.04 (0.73-1.47) | 1.19 (0.86-1.64)  1.35 (0.91-1.99) | 1.14 (0.86-1.49)  1.16 (0.83-1.62) | 1.23 (0.88-1.72)  0.99 (0.67-1.47) |
| Evaluation of students’ academic progress | | | | |
| Medium  High | 1.06 (0.80-1.41)  1.31 (0.97-1.78) | 0.91 (0.67-1.25)  1.33 (0.94-1.88) | 1.00 (0.76-1.30)  1.14 (0.85-1.54) | 0.96 (0.69-1.32)  0.90 (0.63-1.27) |
| Teachers’ expectations for students’ behavior and academic performance | | | | |
| Medium  High | 0.86 (0.63-1.17)  1.08 (0.79-1.48) | 0.97 (0.68-1.39)  1.27 (0.89-1.83) | 0.97 (0.68-1.39)  1.27 (0.89-1.83) | 1.34 (0.93-1.93)  1.15 (0.79-1.66) |
| Communication between school and home | | | | |
| Medium  High | 1.07 (0.80-1.45)  1.41 (0.99-2.02) | 1.03 (0.73-1.45)  1.05 (0.69-1.61) | 1.17 (0.88-1.56)  1.22 (0.85-1.76) | 1.22 (0.87-1.70)  0.97 (0.64-1.46) |
| Perceived teacher agreement about school goals, norms and rules | | | | |
| Medium  High | 0.99 (0.74-1.32)  1.33 (0.96-1-84) | 1.20 (0.87-1.65)  1.37 (0.94-2.00) | 1.17 (0.89-1.54)  1.33 (0.96-1.83) | 1.41 (1.01-1.96)  1.14 (0.79-1.66) |
| Principal’s pedagogic leadership | | | | |
| Medium  High | 1.26 (0.93-1.72)  1.12 (0.81-1.55) | 1.03 (0.72-1.47)  1.24 (0.86-1.80) | 1.33 (0.99-1.80)  1.24 (0.91-1.70) | 0.97 (0.69-1.37)  1.09 (0.77-1.56) |
| Student focus  Medium  High | 1.29 (0.93-1.79)  1.52 (1.08-2.13) | 1.27 (0.88-1.83)  1.44 (0.98-2.11) | 1.25 (0.92-1.71)  1.23 (0.88-1.71) | 0.95 (0.66-1.38)  0.98 (0.67-1.43) |
| Teachers’ perception of school management’s involvement and support | | | | |
| Medium  High | 1.10 (0.81-1.48)  1.34 (0.96-1.87) | 1.16 (0.82-1.63)  1.40 (0.95-2.05) | 1.39 (1.04-1.85)  1.20 (0.86-1.66) | 1.16 (0.82-1.63)  1.11 (0.76-1.61) |
| Teaching activities  Medium  High | 1.19 (0.87-1.62)  1.46 (1.00-2.12) | 1.56 (1.11-2.19)  1.70 (1.10-2.61) | 1.37 (1.02-1.83)  1.62 (1.12-2.35) | 0.88 (0.62-1.24)  0.90 (0.59-1.38) |
| Teacher interaction and cooperation | | | | |
| Medium  High | 1.14 (0.85-1.52)  1.52 (1.12-2.06) | 1.69 (1.21-2.35)  1.82 (1.27-2.61) | 1.18 (0.89-1.55)  1.23 (0.90-1.68) | 1.25 (0.89-1.74)  1.20 (0.84-1.72) |
| Teachers’ confidence and professional development | | | | |
| Medium  High | 1.29 (0.94-1.77)  1.46 (1.03-2.06) | 1.45 (1.02-2.05)  1.46 (0.99-2.16) | 1.44 (1.07-1.93)  1.30 (0.93-1.82) | 1.21 (0.85-1.72)  1.11 (0.75-1.66) |
| Student PESOC |  |  |  |  |
| Expectations  Medium  High | 0.97 (0.72-1.30)  0.91 (0.66-1.26) | 1.08 (0.78-1.47)  1.01 (0.71-1.44) | 1.03 (0.78-1.36)  0.99 (0.73-1.35) | 0.91 (0.65-1.27)  1.14 (0.80-1.62) |
| Perception of teacher norms | | | | |
| Medium  High | 1.01 (0.75-1.35)  1.03 (0.75-1.43) | 1.25 (0.91-1.72)  1.14 (0.79-1.63) | 1.05 (0.80-1.38)  1.05 (0.77-1.43) | 0.97 (0.70-1.36)  0.96 (0.67-1.39) |
| School environment  Medium  High | 1.08 (0.82-1.42)  1.14 (0.82-1.57) | 1.46 (1.08-1.97)  1.78 (1.25-2.53) | 1.28 (0.99-1.65)  1.31 (0.97-1.78) | 1.13 (0.82-1.55)  1.38 (0.97-1.97) |
| School and home  Medium  High | 1.06 (0.78-1.41)  1.13 (0.84-1.52) | 1.09 (0.80-1.50)  1.12 (0.81-1.55) | 0.95 (0.73-1.25)  0.98 (0.74-1.30) | 1.10 (0.80-1.51)  1.12 (0.80-1.57) |
| School management  Medium  High | 1.03 (0.78-1.37)  0.96 (0.70-1.31) | 0.91 (0.66-1.24)  1.25 (0.89-1.76) | 1.05 (0.81-1.37)  1.02 (0.76-1.38) | 0.85 (0.62-1.17)  0.89 (0.63-1.26) |
| Student participation  Medium  High | 1.13 (0.85-1.51)  1.13 (0.84-1.53) | 1.36 (0.98-1.87)  1.43 (1.03-2.00) | 1.31 (1.00-1.73)  1.22 (0.92-1.62) | 0.86 (0.62-1.20)  0.94 (0.68-1.31) |
| Teaching activities  Medium  High | 1.11 (0.83-1.50)  1.11 (0.81-1.53) | 1.24 (0.90-1.72)  1.10 (0.77-1.57) | 1.15 (0.87-1.52)  1.02 (0.75-1.38) | 0.97 (0.69-1.37)  0.93 (0.65-1.33) |
| Teachers’ support  Medium  High | 0.93 (0.70-1.25)  0.99 (0.73-1.34) | 1.19 (0.86-1.63)  1.19 (0.85-1.68) | 1.06 (0.80-1.39)  1.10 (0.83-1.47) | 1.03 (0.74-1.43)  1.00 (0.70-1.41) |

OR, odds ratio; CI, confidence intervals; PESOC, Pedagogical and School Climate Questionnaire; SDQ, Strengths and Difficulties Questionnaire; CES-DC, Center for Epidemiological Studies Depression Scale for Children.

a: The reference category is the first tertile. Adjustment was made for school ownership, the school’s geographical location, adolescent’s gender, CES-DC/SDQ score in grade 7, parental education, parental country of origin, parental cognitive school engagement, percentage of parents with post-high school education, percentage of parents born abroad and percentage of teachers with pedagogical university degree at the school level.

**Table S4** Odds ratios for high parent-reported SDQ according to tertiles of total PESOC score

| Measure of mental health | Poor mental health  (%)^a^  grade 8 grade 9 | | Model 1  OR (95% CI) | Model 2^b^  OR (95% CI) | Model 3^c^  OR (95% CI) |
| --- | --- | --- | --- | --- | --- |
| Teacher PESOC score tertile | | | | | |
| SDQ total problems  Low  Middle  High | 3.70  2.88  3.83 | 2.94  1.80  3.52 | 1.00  0.73 (0.47-1.13)  1.15 (0.77-1.72) | 1.00  0.64 (0.35-1.16)  1.27 (0.71-2.26) | 1.00  0.64 (0.36-1.16)  1.21 (0.70-2.09) |
| SDQ internalizing problems | | | | | |
| Low  Middle  High | 7.09  8.20  7.96 | 6.81  5.63  8.59 | 1.00  0.99 (0.73-1.33)  1.22 (0.91-1.63) | 1.00  0.97 (0.68-1.37)  1.27 (0.89-1.81) | 1.00  0.99 (0.69-1.42)  1.29 (0.90-1.85) |
| SDQ externalizing problems | | | | | |
| Low  Middle  High | 2.11  1.38  2.56 | 2.48  1.80  1.54 | 1.00  0.71 (0.35-1.43)  0.98 (0.48-2.01) | 1.00  0.71 (0.35-1.43)  0.98 (0.48-2.01) | 1.00  0.77 (0.38-1.54)  0.96 (0.49-1.88) |
| Student PESOC score tertile | | | | | |
| SDQ total problems  Low  Middle  High | 3.33  3.66  3.31 | 2.61  2.35  3.30 | 1.00  0.98 (0.65-1.49)  1.17 (0.76-1.80) | 1.00  0.98 (0.57-1.70)  1.46 (0.80-2.64) | 1.00  0.95 (0.56-1.62)  1.42 (0.80-2.51) |
| SDQ internalizing problems | | | | | |
| Low  Middle  High | 7.44  8.68  7.00 | 6.53  6.27  8.11 | 1.00  1.02 (0.77-1.35)  1.05 (0.78-1.41) | 1.00  1.02 (0.73-1.42)  1.12 (0.78-1.60) | 1.00  0.96 (0.68-1.34)  1.05 (0.72-1.51) |
| SDQ externalizing problems | | | | | |
| Low  Middle  High | 1.55  1.88  2.46 | 2.94  1.92  0.76 | 1.00  0.94 (0.57-1.55)  0.88 (0.51-1.50) | 1.00  1.08 (0.57-2.03)  1.24 (0.61-2.54) | 1.00  1.08 (0.57-2.03)  1.24 (0.61-2.54) |

OR, odds ratio; CI, confidence intervals; PESOC, Pedagogical and School Climate Questionnaire; SDQ, Strengths and Difficulties Questionnaire; CES-DC, Center for Epidemiological Studies Depression Scale for Children.

a: Subjects with missing data are excluded.

b: Adjusted for school ownership, the school’s geographical location, adolescent’s gender, CES-DC/SDQ score in grade 7, parental education and parental country of origin.

c: Includes besides the variables in model 2 parental cognitive school engagement, percentage of parents with post-high school education, percentage of parents born abroad and percentage of teachers with pedagogical university degree at the school level.

Figure S1a.


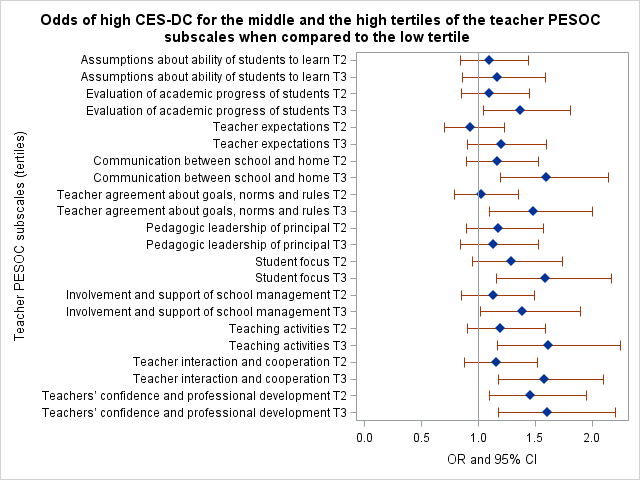


Figure S1b.


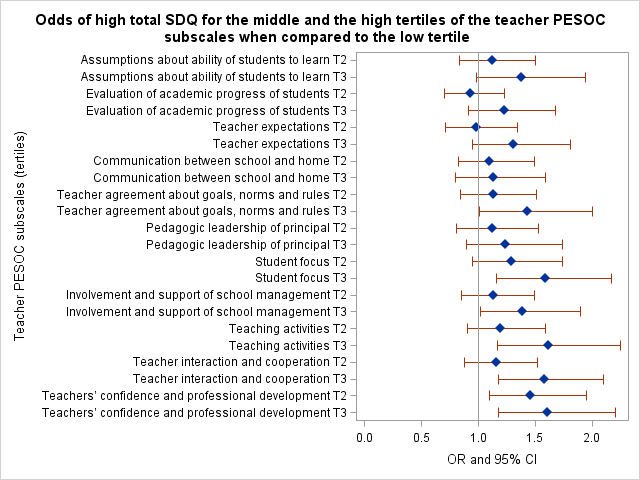


Figure S1c.


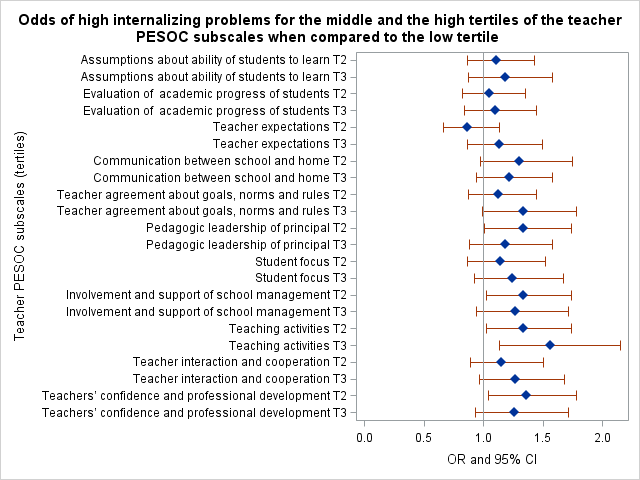


Figure S1d.


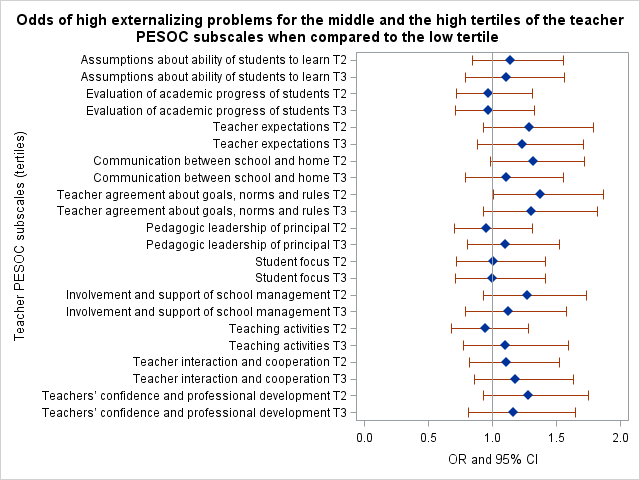


Figure S2a.


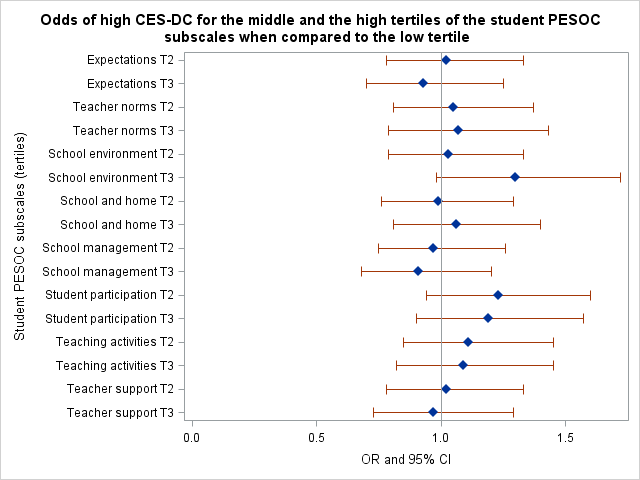


Figure S2b.


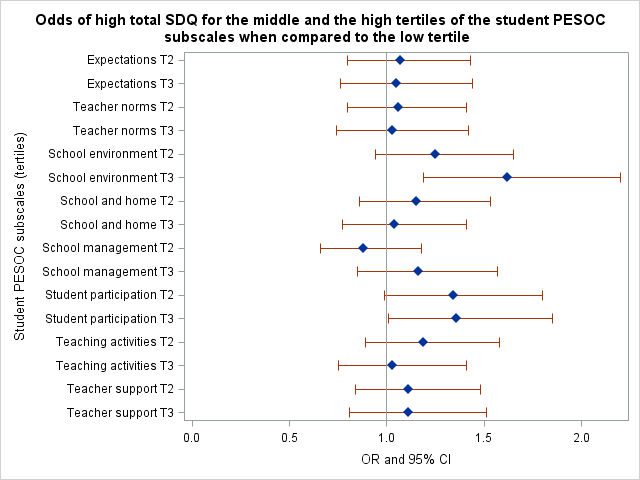


Figure S2c.


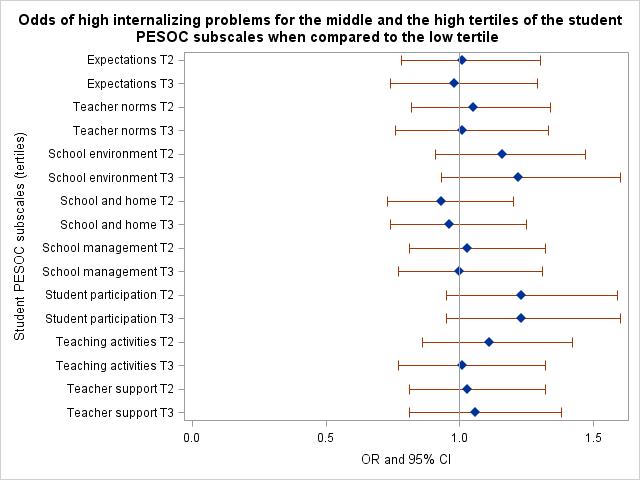


Figure S2d.


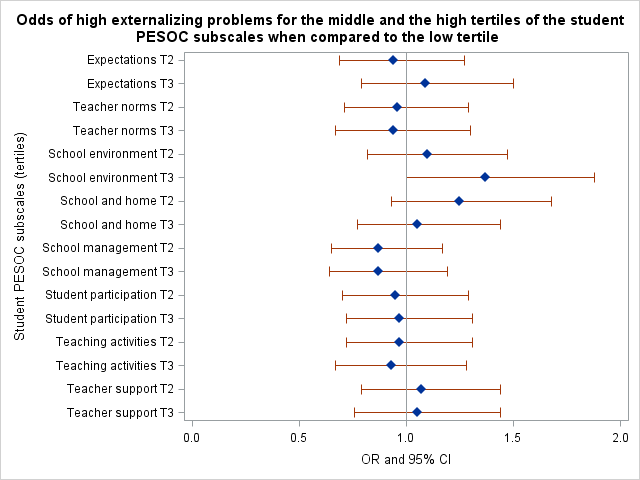

Supplement: Supplementary file 1 — Additional file 1. Supplementary materials. [file 12889_2019_8018_MOESM1_ESM.docx]
